# To What Extent Do Fluorophores Bias the Biological Activity of Peptides? A Practical Approach Using Membrane-Active Peptides as Models

**DOI:** 10.3389/fbioe.2020.552035

**Published:** 2020-09-09

**Authors:** Marco Cavaco, Clara Pérez-Peinado, Javier Valle, Rúben D. M. Silva, João D. G. Correia, David Andreu, Miguel A. R. B. Castanho, Vera Neves

**Affiliations:** ^1^Instituto de Medicina Molecular, Faculdade de Medicina, Universidade de Lisboa, Lisbon, Portugal; ^2^Proteomics and Protein Chemistry Unit, Department of Experimental and Health Sciences, Pompeu Fabra University, Barcelona, Spain; ^3^Centro de Ciências e Tecnologias Nucleares and Departamento de Engenharia e Ciências Nucleares, Instituto Superior Técnico, Universidade de Lisboa, Lisbon, Portugal

**Keywords:** anticancer peptides, BBB peptide shuttles, fluorescence, fluorophore, labeling

## Abstract

The characterization of biologically active peptides relies heavily on the study of their efficacy, toxicity, mechanism of action, cellular uptake, or intracellular location, using both *in vitro* and *in vivo* studies. These studies frequently depend on the use of fluorescence-based techniques. Since most peptides are not intrinsically fluorescent, they are conjugated to a fluorophore. The conjugation may interfere with peptide properties, thus biasing the results. The selection of the most suitable fluorophore is highly relevant. Here, a comprehensive study with blood–brain barrier (BBB) peptide shuttles (PepH3 and PepNeg) and antimicrobial peptides (AMPs) (vCPP2319 and Ctn[15-34]), tested as anticancer peptides (ACPs), having different fluorophores, namely 5(6)-carboxyfluorescein (CF), rhodamine B (RhB), quasar 570 (Q570), or tide fluor 3 (TF3) attached is presented. The goal is the evaluation of the impact of the selected fluorophores on peptide performance, applying routinely used techniques to assess cytotoxicity/toxicity, secondary structure, BBB translocation, and cellular internalization. Our results show that some fluorophores significantly modulate peptide activity when compared with unlabeled peptides, being more noticeable in hydrophobic and charged fluorophores. This study highlights the need for a careful experimental design for fluorescently labeled molecules, such as peptides.

## Graphical Abstract

With this work we highlight the need for a careful experimental design when using fluorescently labeled molecules, since we show that some fluorophores significantly modulate peptide activity, which may contribute to biased results.

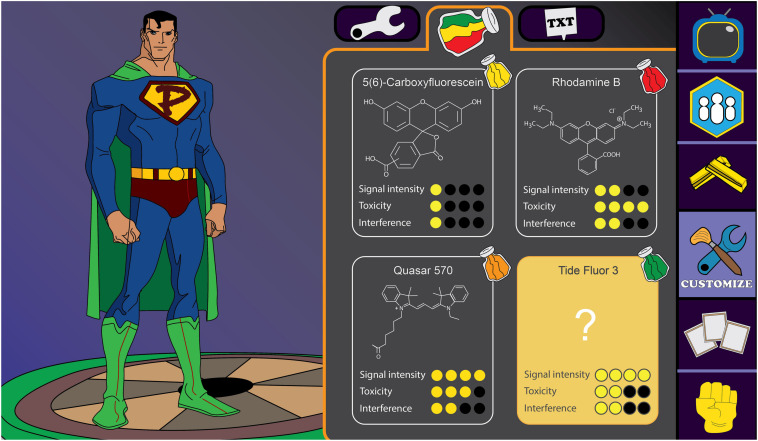

## Introduction

The development of molecules for biological or biomedical applications relies on their accurate and precise biophysical/biological characterization (La Gatta et al., 2016; Van Norman, 2016). The necessary data concerning the efficacy, toxicity, mechanism of action, cellular uptake, or intracellular location of such molecules can be gathered using *in vitro* or *in vivo* approaches (D’Addio et al., 2016). The collection of this information requires the use of highly sensitive techniques, which usually depend on the use of fluorescent probes (Gonzalez-Vera, 2012; Xu et al., 2018; Gao and Wu, 2019). Since most molecules are not intrinsically fluorescent in the visible spectrum range, conjugation to a fluorophore is needed. This way, fluorescence-based techniques, such as confocal laser scanning microscopy (CLSM), flow cytometry, and fluorimetry are possible options (Gautam et al., 2015; Radicioni et al., 2015). Despite their extensive use and value, most fluorophores are bulky, rigid, and hydrophobic molecules. Hence, their conjugation to other molecules may alter their physicochemical/biological properties, mainly when dealing with low molecular weight molecules, which may ultimately bias the results obtained by a given technique (Toseland, 2013; Sánchez-Rico et al., 2017; Hedegaard et al., 2018).

The selection of the fluorophore is usually based on chemical intuition, cost, and photophysical properties. The major parameters considered in the selection of a fluorophore are the excitation/emission wavelength, brightness, photobleaching, photostability, chemical reactions required, or conjugation yields. The assumption that unlabeled- and labeled-molecules have the same properties often relies on wish much more than evidence (Zhao et al., 2016). The advent of studies reporting differences between fluorophores raised awareness to the importance of their selection (Fischer et al., 2002; Toseland, 2013; Knutson et al., 2016; Zhao et al., 2016; Birch et al., 2017; Hedegaard et al., 2018). In addition, some studies also report the impact of the molecule on the properties of the fluorophore (Toseland, 2013; Szabó et al., 2018). The choice of the most suitable fluorophore to label molecules of interest is thus of utmost importance, not compatible with unworthy presumptions.

In the last decades, the interest in peptide-based systems has increased owing to improvements in knowledge and manipulation of peptide physicochemical properties (Fosgerau and Hoffmann, 2015; Boone et al., 2018; Ghasemy et al., 2018). As a result, peptides are now part of different therapeutic/diagnostic protocols. The main applications in therapeutic medicine are in cancer [anticancer peptides (ACPs)] and infectious diseases [antimicrobial peptides (AMPs)] (Gaspar et al., 2013; Shoombuatong et al., 2018). Radiolabeled-peptides for targeted nuclear molecular imaging and/or systemic radiotherapy play unique roles in nuclear medicine and oncology (Correia et al., 2011; Oliveira and Correia, 2019). Additionally, peptides have also been employed in drug-delivery systems for their receptor specificity and cargo translocation capacity across either epithelial or endothelial cellular barriers (Zou et al., 2013; Oller-Salvia et al., 2016; Gallo et al., 2019). Like many other molecules, peptides are not intrinsically fluorescent in the visible range of the electromagnetic spectrum. Thus, they are a good example of a molecule of therapeutic and biological interest that must be conjugated to a fluorophore. Recently, unnatural fluorescent amino acids have been tested as an alternative to the use of fluorophores (Mendive-Tapia et al., 2016; Cheng et al., 2020; Subiros-Funosas et al., 2020), presenting several advantages over fluorophores. Nevertheless, the use of fluorophores is still broadly used to investigate peptide biological activity.

The aim of the present work was the evaluation of the impact of different fluorophores on the properties of peptides having different activities to rationalize the selection of the most adequate fluorophore for a specific application. In contrast to other studies, we selected four model fluorophores commercially available, namely, 5(6)-carboxyfluorescein (CF), rhodamine B (RhB), quasar 570 (Q570), and tide fluor 3 (TF3). This selection covers a broad range of chemical properties of fluorophores. CF is the fluorophore with the lowest molecular weight (376.32 Da), has a medium hydrophobicity, and a negative charge. RhB and Q570 present almost the same size (around 470.0 Da), high hydrophobicity, but different charges. RhB is a neutral molecule, whereas Q570 is positively charged. Finally, TF3 has the highest molecular weight (559.7 Da), high hydrophobicity, and a neutral charge. These fluorophores own a broad range of physicochemical properties (Supplementary Table S1) and were conjugated to PepH3 and PepNeg, which are blood–brain barrier (BBB) peptide shuttles; and vCPP2319 and Ctn[15-34], which are AMPs and were tested as ACPs in this study (Supplementary Tables S2, S3). To the best of our knowledge, no studies have been published reporting the differential effect of fluorophores on bioactive peptides according to the functional classes they belong to. The effect of fluorophores were only tested, in separate studies, in AMPs (Zhao et al., 2016) or cell-penetrating peptides (CPPs) (Fischer et al., 2002; Birch et al., 2017). It is also worth highlighting that, concerning peptide selection, both BBB peptide shuttles have equal length: seven amino acid residues. PepH3 is cationic and has high extinction coefficient, isoelectric point, and hydrophobicity (Neves et al., 2017), whereas on the other hand, PepNeg is an anionic peptide with a low extinction coefficient, isoelectric point, and hydrophobicity (Neves-Coelho et al., 2017). vCPP2319 and Ctn[15-34] are both cationic peptides of twenty amino acid residues having antimicrobial activity (Dias et al., 2017; Pérez-Peinado et al., 2018). Some AMPs can act as ACPs (Gaspar et al., 2013; Felício et al., 2017), thus we assessed the efficacy of vCPP2319 and Ctn[15-34] against cancer cells. All conjugations took place at the N-terminus of the sequence, after selective removal of the Fmoc Na protecting group.

To broaden the comprehensiveness of the study, we studied the peptide secondary structure, cellular uptake, intracellular location, and toxicity to red blood cells (RBCs). Also, *in vitro* BBB translocation assay for BBB peptide shuttles and cytotoxicity toward cancer cells of the last two peptides was carried out. Ultimately, we have concluded on the biasing each fluorophore cause on structural and functional data accounting to the methodologies applied in the experimental assays.

## Materials and Methods

### Chemicals and Materials

Fmoc-protected amino acids, Fmoc-Rink amide (MBHA) resin, 2-(1H-benzotriazol-1-yl)-1, 1,3,3-tetramethyluronium hexafluorophosphate (HBTU), and N-hydroxybenzotriazole (HOBt) were from Iris Biotech (Marktredwitz, Germany). HPLC-grade acetonitrile (ACN), and peptide-synthesis grade N,N-dimethylformamide (DMF), dichloromethane (DCM), N,N-diisopropylethylamine (DIEA), N,N-diisopropylcarbodiimide (DIPCI), trifluoroacetic acid (TFA), and triisopropylsilane (TIS) were from Carlo Erba-SDS (Sabadell, Spain). 3,6-dioxa-1,8-octanedithiol (DODT), CF and RhB were from Sigma-Aldrich (Spain). Q570 was from BioSearch^TM^ Technologies (Spain). TF3 was from AAT Bioquest^®^ (United States).

Dulbecco’s Modified Eagle Medium (DMEM), DMEM/Ham’s F-12 (DMEM:F12), DMEM:F12 without phenol-red, Trypsin-EDTA, attachment factor protein solution (AF), fetal bovine serum (FBS), penicillin-streptomycin (Pen-Strep), and fluorescent dyes Hoechst 33342 were from Life Technologies (Carlsbad, CA, United States).

### Peptide Synthesis and Purification

PepH3 (AGILKRW-amide), PepNeg (SGTQEEY-amide), vCPP2319 (WRRRYRRWRRRRRWRRRPRR-amide), and Ctn[15-34] (KKRLKKIFKKPMVIGVTIPF-amide) were synthetized in a Prelude Synthesizer (Gyros Protein Technologies, Tucson, AZ, United States) running Fmoc (FastMoc) SPPS protocols at 0.1 mmol scale on a Fmoc-Rink-amide ChemMatrix resin (Supplementary Table S3). Side chain functionalities were protected with tert-butyl (Glu, Ser, Thr, Tyr), NG-2,2,4,6,7-pentamethyldihydrobenzofuran-5-sulfonyl (Arg), Nα-tert-butoxycarbonyl (Trp), and trityl (Cys) groups. Eight-fold excess of Fmoc-L-amino acids and HBTU, in the presence of a double molar amount of DIEA, were used for the coupling steps, with DMF as the solvent. After chain assembly, full deprotection and cleavage were carried out with TFA/H_2_O/DODT/TIS (94:2.5:2.5:1 v/v, 90 min, r.t.). The labeled-peptides were similarly synthetized (Supplementary Table S3). However, before the deprotection of all amino acid residues, CF, RhB, Q570, and TF3 were coupled manually with four-fold excess in the presence of an equivalent amount of DIPCDI in DMF. After synthesis completion, peptides were fully deprotected and cleaved with TFA/H_2_O/DODT/TIS (94:2.5:2.5:1 v/v, 90 min, r.t.). Peptides were isolated by precipitation with cold diethyl ether and centrifugation at 4,000 × *g*, 4°C for 20 min. Then, they were taken up in H_2_O and lyophilized.

Analytical reversed-phase HPLC was performed on a Luna C18 column (4.6 mm × 50 mm, 3 μm; Phenomenex, United States). Linear gradients of solvent B (0.036% TFA in MeCN) into solvent A (0.045% TFA in H_2_O) were used at a flow rate of 1 mL/min and with UV detection at 220 nm. Preparative HPLC runs were performed on a Luna C18 column (21.2 mm × 250 mm, 10 μm; Phenomenex) using linear gradients of solvent B (0.1% TFA in MeCN) into solvent A (0.1% TFA in H_2_O) at a flow rate of 25 mL/min and with UV detection at 220 nm. Fractions of adequate HPLC homogeneity and with the expected mass were combined and lyophilized. LC-MS was performed in a LC-MS 2010EV instrument (Shimadzu, Kyoto, Japan) fitted with an XBridge C18 column (4.6 mm × 150 mm, 3.5 μm; Waters, Spain), eluting with linear gradients of HCOOH/MeCN (0.08% v/v) into HCOOH/H_2_O (0.1% v/v) over 15 min at 1 mL/min. Peptide stock solutions (1 mM) in filtered H_2_O were stored at −20°C.

### Measurement of Spectral Properties

Spectroscopic data were recorded on an FS900 fluorometer (Edinburgh Instruments, United Kingdom). The different peptides were dissolved at concentrations around 10–50 μM in 1× PBS and spectra recorded at r.t. (Supplementary Figures S1–S4).

### Circular Dichroism

Circular dichroism (CD) spectra of the different peptides were acquired in a J-815 spectropolarimeter (Jasco, Japan) at 25°C in the 190–260 nm wavelength range, with a bandwidth of 1 nm and a scan speed of 50 nm/min, using a 0.1 cm quartz cell. 50 μM peptide solutions were prepared in 10 mM sodium phosphate (75.4 mM Na_2_HPO_4_, 24.6 mM NaH_2_PO_4_, pH 7.4). The final spectra for each peptide were the average of three consecutive scans per sample after subtraction of buffer baselines (Supplementary Figure S5). Results were expressed as mean residue ellipticity ([θ]_MRW_) (deg × cm^2^ × dmol^–1^), as follows:

(1) $${\left[\theta \right]}_{\text{MRW}}=\frac{\theta_{\text{Obs}}\times \text{MRW}}{10dc}$$

where, θ_obs_ is the observed ellipticity in degrees, MRW is the mean residue weight, d is the cell path length and c is the peptide molar concentration.

### Hemolytic Activity

Fresh human blood was collected in EDTA tubes and centrifuged at 1,000 × *g* for 10 min at 4°C. The supernatant was discharged, and the pellet containing RBCs was washed three times with 1× PBS (137 mM NaCl, 2.7 mM KCl, 10 mM Na_2_HPO_4_, and 1.8 mM KH_2_PO_4_, pH 7.4) and resuspended in 1× PBS to obtain a 2.0% (v/v) suspension. Then, RBCs were added to centrifuge tubes containing two-fold serially diluted peptides to a final concentration ranging from 0.01 to 100 μM. The suspension was incubated for 24 h at 37°C with gentle stirring. After that, samples were centrifuged for 2 min at 1,000 × *g*. Supernatants were transferred to 96-well plates, and the hemoglobin released measured by absorbance at 570 nm in an Infinite F200 TECAN plate reader. 1× PBS with no peptides and Triton X-100 at 1 and 4% (v/v) were used as negative and positive controls, respectively. Hemolytic activity (%) was determined using the following equation:

(2) $$\mathrm{Hemolytic}\mathrm{activity}\left(\%\right)=\frac{{\text{Abs}}_{\text{PT}}-{\text{Abs}}_{\text{NC}}}{{\text{Abs}}_{\text{PC}}-{\text{Abs}}_{\text{NC}}}$$

where, Abs_PT_ is the absorbance of treated samples, Abs_NC_ is the absorbance from negative control, and Abs_PC_ absorbance from positive control.

HC_50_ values were determined using the GraphPad Prism 7.0 software using a log(inhibitor) vs. normalized response. Experiments were performed on different days using independent blood donors.

### Cell Culture

Human cerebral microvascular endothelial cells (HBEC-5i, ATCC^®^ CRL-3245) and Human-breast cancer cell line MDA-MB-231 (ATCC^®^ HTB-26) were purchased from American Type Culture Collection (ATCC, United States). HBEC-5i cells were cultured as a monolayer on 0.1% gelatin solution (Gibco/Thermo Fisher, United States) coated T-flasks in DMEM:F12 medium (Gibco/Thermo Fisher, United States) supplemented with 10% FBS (Gibco/Thermo Fisher, United States), 1% penicillin/streptomycin antibiotic solution (Gibco/Thermo Fisher, United States), and 40 μg/mL endothelial growth supplement (ECGS) (Sigma-Aldrich, Spain), according to the manufacturer’s instructions. MDA-MB-231 cells were cultured as a monolayer in DMEM medium (Gibco/Thermo Fisher, United States) supplemented with 10% FBS and 1% penicillin/streptomycin antibiotic solution, according to manufacturer’s instructions. Both cells were grown in a humidified atmosphere of 5% CO_2_ at 37°C (MCO-18AIC (UV), Sanyo, Japan) with the medium changed every other day.

### *In vitro* Translocation Studies

**FIGURE 1:**
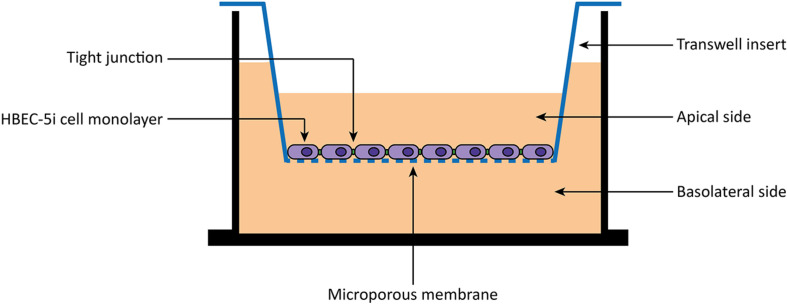
Scheme of an *in vitro* BBB transwell membrane system. Human brain endothelial cells (HBEC-5i) are cultured as monolayer on the apical side of a polyester (PET) transwell insert (1.0 μm pore size). Then, 10 μM of peptides are added to the apical side, and the translocation measured as the amount detected on the basolateral side.

HBEC-5i cells were allowed to grow until confluence in a gelatin-coated T-flask. Then, cells were carefully harvested with trypsin-EDTA (Gibco/Thermo Fisher, United States) and seeded 8,000 cells/well to 0.1% gelatin solution coated tissue culture inserts [transparent polyester (PET) membrane with 1.0 μm pores] for 24-well plates (BD Falcon, United States). During 8 days, the medium was changed every 2 days. After 8 days, cells were washed two times with 1× PBS and once with DMEM:F12 medium without phenol red (Gibco/Thermo Fisher, United States). Then, peptides diluted in DMEM:F12 medium without phenol red to a final concentration of 10 μM were added to the apical side of the *in vitro* BBB model (Figure 1). Experiments were performed on different days using independently grown cell cultures.

### Evaluation by Fluorescence Emission

The translocation of peptides labeled with different fluorophores was determined by fluorescence emission. After 24 h incubation, samples from the apical and basolateral side were collected and analyzed. Fluorescence was measured using the infinite F200 TECAN plate reader. The percentage (%) of translocation was calculated using the following equation:

(3) $$\text{Translocation}\left(\%\right)=\left(\frac{F_{\text{i}}-F_{\text{cells}}}{F_{\text{peptide}}-F_{\text{Medium}}}\right)\times 100$$

*F*_i_ is the fluorescence intensity recovered, *F*_cells_ is the fluorescence intensity recovered from cells without peptide incubation, *F*_peptide_ is the fluorescence intensity of total peptide initially added to the transwell apical side, and *F*_Medium_ is the fluorescence intensity of the medium.

### Evaluation by HPLC

The translocation of unlabeled peptides was determined by the calculation of the respective area under the curve (AUC) in the reversed phase HPLC chromatograms. After 24 h incubation, samples from the apical and basolateral side were collected and analyzed by HPLC on a PerkinElmer Series 200 pump coupled to a PerkinElmer Series 200 UV/Vis Detector on a Luca C18 column (4.6 mm × 50 mm, 3 μm, Phenomenex, United States), eluting with linear gradients of HCOOH/MeCN (0.1%, v/v) into HCOOH/H_2_O (0.1%, v/v) over 15 min at 1 mL/min. UV detection was λ = 220 nm. The percentage (%) of translocation was calculated using the following equation:

(4) $$\text{Translocation}\left(\%\right)=\left(\frac{{\text{AUC}}_{\text{i}}}{{\text{AUC}}_{\text{peptide}}}\right)\times 100$$

AUC_i_ is the AUC of peptide recovered and AUC_peptide_ is the AUC of total peptide initially added to the transwell apical side.

### *In vitro* Integrity Assay

After the 24 h incubation period with the peptides, an *in vitro* integrity assay was performed. Herein, cells were washed two times with 1× PBS and once with DMEM:F12 medium without phenol. Then, previously diluted fluorescein isothiocyanate-dextran with an MW of 4 kDa (FD4) (Sigma-Aldrich, Spain) was added to the apical side and incubated for 2 h. FD4 was diluted in DMEM:F12 medium without phenol to an absorbance of 0.1. Samples were collected from the apical and basolateral side, and fluorescence intensity was measured at λ with an excitation of 493 nm and maximum emission at 560 nm using the infinite F200 TECAN plate reader. The percentage of FD4 recovered was determined using the following equation:

(5) $$\mathrm{FD4}\mathrm{Permeability}\left(\%\right)=\left(\frac{F_{\text{i}}-F_{\text{cells}}}{F_{\mathrm{FD4}}-F_{\text{Medium}}}\times 100\right)$$

*F*_i_ is the fluorescence intensity recovered, *F*_cells_ is the fluorescence intensity recovered from cells without FD4 incubation, *F*_FD__4_ is the fluorescence intensity of total FD4 initially added to the transwell apical side, and F_Medium_ is the fluorescence intensity of DMEM:F12 medium without phenol red.

The integrity of the barrier is indirectly proportional to the percentage of FD4 recovered and was determined using the following equation:

(6) Integrity(%)=100-FD4Permeability(%).

### Cell Viability Measurements

Peptides cytotoxicity against both HBEC-5i and MDA-MB-231 cells was determined using CellTiter-Blue^®^ Cell Viability Assay, according to the manufacturer’s instructions. The assay is based on the ability of viable cells to reduce resazurin into resorufin, a highly fluorescent metabolite. On the other hand, non-viable cells rapidly lose metabolic capacity and, consequently, do not generate resorufin. Therefore, using this methodology is possible to distinguish between metabolic and non-metabolic cells and indirectly determine the cytotoxicity of peptides. Briefly, HBEC-5i and MDA-MB-231 cells were carefully detached from T-flasks, as described previously, and seeded 15,000/100 and 10,000/100 μL, respectively, in 96-well plates (Corning, United States) and incubated for 24 h. After medium removal, cells were washed two times with 1× PBS and 100 μL of previously diluted peptides (range between 0.01 and 100 μM) in either DMEM or DMEM:F12 medium were added to MDA-MB-231 or HBEC-5i cells, respectively. After 24 h incubation, cells were washed two times with 1× PBS, and 20 μL of CellTiter-Blue^®^ Reagent (diluted in 100 μL of medium) was added to each well and incubated for 3 h in a humidified atmosphere of 5% CO2 at 37°C. The fluorescence intensity was measured at λ with an excitation of 560 nm and maximum emission at 590 nm using the infinite F200 TECAN plate reader. Medium and 1% Triton X-100-containing medium were used as positive controls (100% cell viability) and negative controls (0% cell viability), respectively. Cell viability (%) was determined using the following equation:

(7) $$\mathrm{Cell}\,\mathrm{Viability}=\frac{F_{\text{P}}-F_{\text{NC}}}{F_{\text{PC}}-F_{\text{NC}}}\times 100$$

*F*_p_ is the fluorescence intensity of peptide-treated cells, *F*_NC_ is the fluorescence intensity for negative controls, and *F*_PC_ is the fluorescence intensity for positive controls.

IC_50_ values were determined using the GraphPad Prism 7.0 software using a log(inhibitor) vs. normalized response. Experiments were performed on different days using independently grown cell cultures.

### Confocal Microscopy

HBEC-5i and MDA-MB-231 cells were seeded 50,000/200 μL on an ibiTreat-coated 8-well μ-slide (Ibidi, Germany) for 24 h. Then, cells were washed carefully two times with 1× PBS and once with medium and incubated for 2 h with labeled peptides at a final concentration of 10 μM. Nucleus was stained with Hoescht 33342 (Thermo Fisher, United States). After cell washing, nucleus dye was added to cells at a final concentration of 5 μg/mL and for 10 min at 37°C. Finally, cells were washed twice with 1× PBS and imaged.

The acquisition was made on a confocal point-scanning Zeiss LSM 880 microscope (Carl Zeiss, Germany) equipped with an alpha Plan-Apochromat X 63 oil immersion objective (1.40 numerical aperture). Diode 405-30 laser was used to excite Hoechst 33342 (Sigma-Aldrich, Spain). The 488 nm line from an argon laser was used to excite peptide labeled with CF and NeNe594 laser was used to excite peptides labeled with RhB, Q570, and TF3. In the normal confocal mode, X 0.6 zoom images were recorded at 2048 × 2048 resolution. ZEN software as used for image acquisition. Fiji software was used for image processing. At least 12 total images were acquired in three independent replicates.

To compare the fluorescence intensities of the different fluorophores within different cells, we calculated the corrected total cell fluorescence (CTCF) using the following equation:

(8) CTCF=IntegratedDensity-(Areaofselectedcell×Meanfluorescenceofbackgroundreadings)

### Statistical Analysis

Quantitative data were processed using Excel 2013 (Microsoft, United States) and the GraphPad Prism 7.0 software package. Medians, means and standard deviations are shown in the figures and tables. Pairwise significances were calculated using one-way ANOVA followed by Tukey’s multiple comparison test, and non-parametric Mann–Whitney, Kruskal–Wallis.

## Results and Discussion

We have selected four different fluorophores (CF, RhB, Q570, and TF3) to evaluate their impact on the characterization of peptides. Two families of peptides were used: BBB peptide shuttles (PepH3 and PepNeg), and AMPs (vCPP2319 and Ctn[15-34]), which were tested as ACPs in this study since many AMPs have anticancer properties. Both labeled- and unlabeled-peptides were thoroughly characterized regarding structure using CD; toxicity toward RBCs and endothelial cells; translocation in a BBB *in vitro* model; and anticancer activity in a triple-negative breast cancer (TNBC) cell line. The cellular uptake of the peptides was determined by confocal microscopy.

### Toxicity to Human Erythrocytes

The characterization of new biomolecules, such as peptides, includes the evaluation of their toxicity. The information collected helps avoiding rejection in preclinical stages, for instance (Hughes et al., 2011; Lau and Dunn, 2018). A standard evaluation of safety is to determine toxicity toward human RBCs since hemolytic assays are easy to perform, robust, cheap, and highly informative (Colonna et al., 2017).

**FIGURE 2:**
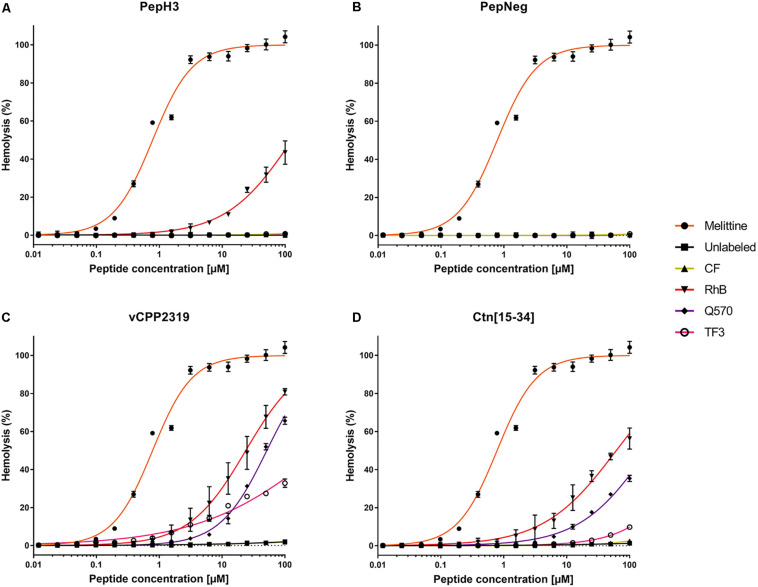
Interaction of unlabeled- and labeled-peptides with RBCs. A suspension of RBCs (1.0%, v/v) was incubated with various concentrations of **(A)** PepH3, **(B)** PepNeg, **(C)** vCPP2319, and **(D)** Ctn[15-34] (range from 0.01 to 100 μM). The percentage of hemolysis was determined by the absorbance of hemoglobin released into the supernatant. Melittin was included as a control peptide with hemolytic properties. The values were obtained from triplicates of three independent experiments. Error bars, S.D.

In the present work, we evaluated the tendency of both labeled- and unlabeled-peptides to induce hemolysis in freshly collected RBCs. The results reveal differential hemolytic activity concerning the fluorophores and peptides employed (Figure 2). The peptides showing the lowest hemolytic activity are PepH3 and PepNeg. Unlabeled-PepH3 is non-hemolytic up to 100 μM (Figure 2A and Supplementary Table S4). Upon derivatization, only RhB-PepH3 shows a significantly increased toxicity (HC_10_ = 9.208 μM, and HC_50_ = 128.3 μM) (Figure 2A and Supplementary Table S4), while PepNeg has non-hemolytic activity up to 100 μM (Figure 2B and Supplementary Table S4) for both unlabeled- and labeled forms. On the other hand, vCPP2319 and Ctn[15-34] show a different hemolytic profile. In both cases, unlabeled- and CF-peptides are non-hemolytic up to 100 μM. Contrariwise, conjugation to RhB, Q570, and TF3 demonstrates a significantly increased toxicity (Figures 2C,D and Supplementary Table S4). Since the most common mechanism of action of AMPs corresponds to membrane disruption, these findings are in agreement with the high affinity of these peptides toward lipid membranes (Hoskin and Ramamoorthy, 2008). Nevertheless, the toxicity increases with their conjugation to highly hydrophobic fluorophores (RhB and Q570).

The hemolysis rate in RBCs exposed to labeled-peptides show that CF-peptides do not exert damaging effects in comparison to other derivatives. Overall, all other labeled-peptides exhibit low to moderate hemolysis (TF3-peptide) or pronounced hemolysis (RhB-peptide and Q570-peptide). The correlation of this finding to the structure of fluorophores shows that negatively charged fluorophores contribute the least to membrane damaging (CF-peptide). By contrast, we observe more severe hemolysis with the conjugation to fluorophores that increase net charge compared with unlabeled-peptides, i.e., RhB-peptide, Q570-peptide, and TF3-peptide. Moreover, the combination of increased positive net charge and high hydrophobicity observed with RhB-peptides and Q570-peptides expand the hemolytic properties of unlabeled-peptides. This observation supports the idea that cationic and hydrophobic derivatives can disrupt membranes in a more efficient way than peptides conjugated to neutral or anionic fluorophores, which is in line with previous works (Birch et al., 2017; Avci et al., 2018). Thus, the outcome of a hemolytic assessment is highly dependent on the fluorophore-conjugated.

### Translocation and Toxicity Across an *in vitro* BBB Model

The BBB is a physiological barrier responsible for the maintenance of the brain homeostasis (Weiss et al., 2009; Serlin et al., 2015). Therefore, strategies to overcome the BBB are an unmet clinical need (Hersh et al., 2016; Wang et al., 2019). Among others, the conjugation of therapeutics to BBB peptide shuttles has been one of the most promising (Oller-Salvia et al., 2016; Sánchez-Navarro et al., 2017). In addition, a good *in vitro* BBB model is also fundamental for a proper characterization of the translocation properties of the conjugates. Although different models have been described, (Helms et al., 2016) our lab optimized the use of monoculture BBB models. Thus, in a quick and robust way, we can easily access the translocation capabilities of many BBB peptide shuttles. Among the different cell lines that can be used within the model, (Helms et al., 2016) in the present study, we have successfully used HBEC-5i cells (Figure 1).

To assess the effect of the fluorophore on the translocation properties of BBB peptide shuttles, we selected two different peptides. PepH3 is a seven amino acid peptide derived from the α3-helical domain of the Dengue virus capsid protein (DEN2C) (Neves et al., 2017). It is a cationic and hydrophobic peptide able to translocate endothelial membranes *in vitro* and *in vivo* (Côrte-Real et al., 2016; Neves et al., 2017). PepNeg is a new anionic peptide designed based on the sequence of PepH3 (Neves-Coelho et al., 2017). Our *in vitro* data also demonstrates that PepNeg efficiently transports cargo through the *in vitro* BBB model without disrupting the barrier (Neves-Coelho et al., 2017).

**FIGURE 3:**
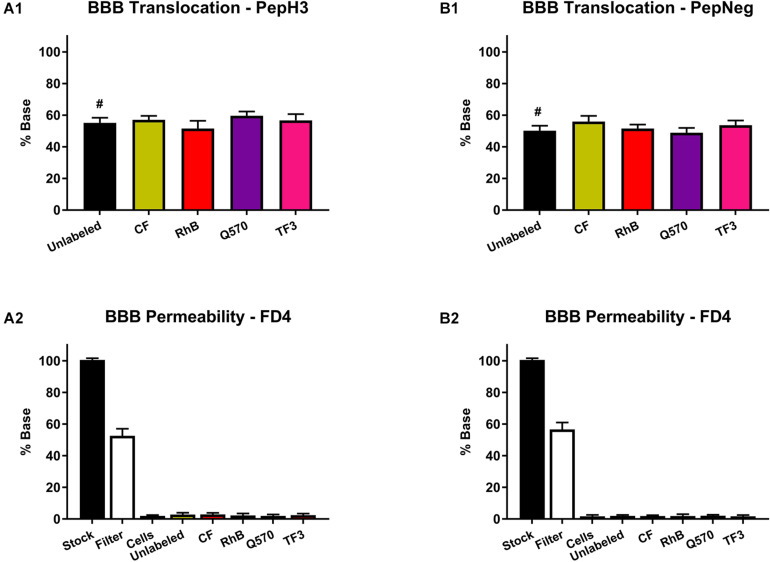
Translocation of peptide derivatives across an *in vitro* BBB model and the FD4 permeability study. **(A1)** Percentage of translocation of PepH3 (10 μM). **(A2)** Fluorescence intensity of FD4 measured after translocation assay with PepH3. **(B1)** Percentage of translocation of PepNeg (10 μM). **(B2)** Fluorescence intensity of FD4 measured after translocation assay with PepNeg. The values were obtained from triplicates of three independent experiments. # represents the quantification using an HPLC. Statistical significance analysis was evaluated with a one-way ANOVA followed by Tukey’s multiple comparison test and no statistical significance difference was observed between samples.

Within this study, results show that both PepH3 and PepNeg translocate the HBEC-5i *in vitro* BBB model in an efficient way (Figures 3A1,B1). For the labeled-peptides, we performed the quantification of the translocation percentage by measuring the fluorescence intensity in the basolateral side. Concerning unlabeled-peptides, we performed the quantification by HPLC. In all cases, the translocation (%) was above 50%, which is in line with previous results (Neves et al., 2017; Neves-Coelho et al., 2017). Thus, this data suggests that none of the fluorophores has an impact on the translocation capacity of both BBB peptide shuttles. Next, we assessed the endothelial barrier integrity of the *in vitro* BBB model by the fluorescence intensity of FD4 recovered (Figures 3A2,B2). None of the unlabeled- or labeled-peptides influence the barrier permeability (HBEC-5i integrity > 95%). This finding also suggests that none of the derivatives is toxic toward HBEC-5i cell line.

**FIGURE 4:**
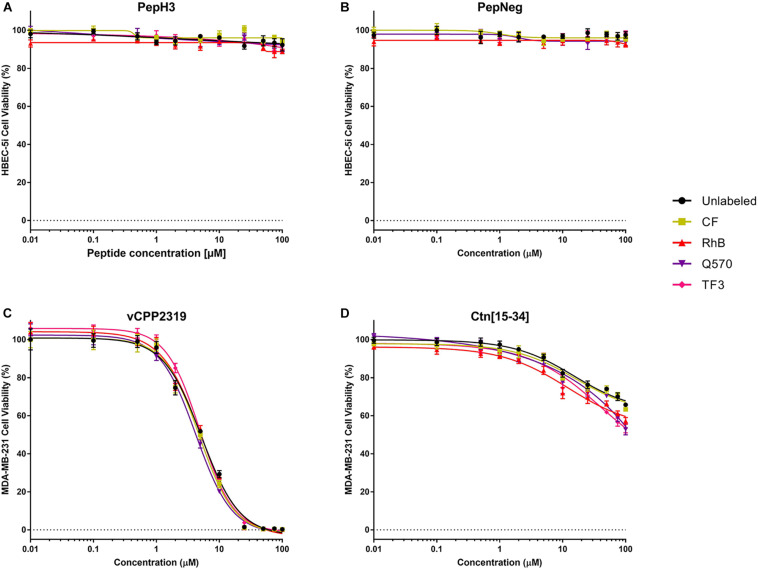
*In vitro* cytotoxicity unlabeled- and labeled-peptides. **(A,B)** Cytotoxicity against HBEC-5i cells. **(C,D)** Cytotoxicity against MDA-MB-231 cells. 1.5 × 10^4^ and 1.0 × 10^4^ cells of HBEC-5i and MDA-MB-231 cells, respectively, were incubated with various concentrations of peptides (range from 0.01 to 100 μM). The percentage of viability was determined using CellTiter^®^ Blue reagent assay. The values were obtained from triplicates of three independent experiments. Error bars, S.D.

In addition, we also performed a cell viability assay to confirm the absence of toxicity. For all the unlabeled- and labeled-peptides, the IC_50_ is always > 200 μM. Nevertheless, the labeling to more hydrophobic fluorophores, such as TF3, Q570, or RhB, seems to increase cell death. Even so, in both assays, all peptides show no significant toxicity toward HBEC-5i cells (Figures 4A,B and Supplementary Table S5).

### Anticancer Activity in Breast Cancer Cells

The anticancer activity of vCPP2319 and Ctn[15-34], as well as their derivatives, was evaluated in MDA-MB-231 cells, since it is widely used as a TNNC cell model (Liu et al., 2019). We used the reduction of resazurin into resorufin as viability assay, and the fluorescence of the metabolite was measured using a plate reader.

The cytotoxicity results are shown in Figures 4C,D and Supplementary Table S5. Unlabeled- and labeled-vCPP2319 show an IC_10_ between 1.1 and 1.3 μM, an IC_50_ between 4 and 5 μM, and an IC_90_ between 16.0 and 20.6 μM, respectively. Q570-vCPP2319 has the lowest values (IC_10_ = 1.14 μM, IC_50_ = 4.26 μM, and IC_90_ = 16.0 μM). Although the values do not change significantly, the analysis of the IC_90_ demonstrates that the labeling to more hydrophobic fluorophores (Q570 and TF3) slightly increases toxicity. On the other hand, the effect of Ctn[15-34] is different. The IC_50_ of all peptides is >100 μM, which demonstrates that the peptide does not have activity toward MDA-MB-231 cells. Nevertheless, Q570-Ctn[15-34] and TF3-Ctn[15-34] presented an IC_50_ of 144.0 and 122.7 μM, respectively. Although no important differences are observed with the IC_50_, the analysis of the IC_10_ demonstrates that the conjugation of Ctn[15-34] to RhB, Q570, and TF3 increases the cytotoxicity of the peptide. Ctn[15-34] demonstrates no anticancer properties toward MDA-MB-231 cell line. However, the peptide was studied in parallel with vCPP2319 for comparative purposes. Therefore, the results reported in this assay are also sensitive to the fluorophore-conjugated.

### Cellular Imaging of Labeled Peptides

The use of microscopy techniques in the characterization of peptides is a common practice. The results obtained allowed us to evaluate the capacity of the peptide to be internalized, the specificity of peptides toward some cell lines, the internalization mechanism, and the possible mechanism of actions, for instance (Shen et al., 2007; Matsumoto et al., 2015; Rezgui et al., 2016; Kumar et al., 2018). Herein, we assessed the uptake of both BBB peptide shuttles on HBEC-5i cells and both AMPs on MDA-MB-231 cells.

**FIGURE 5:**
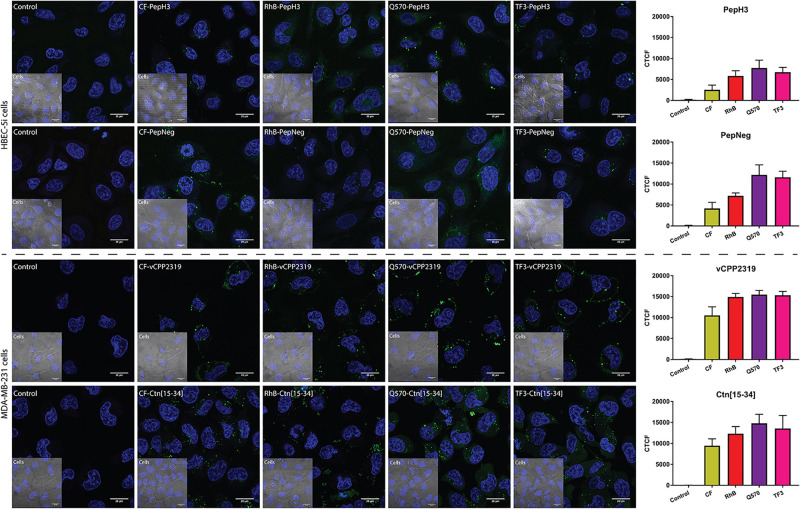
Representative confocal microscopy images of peptide internalization. Confocal microscopy analysis of HBEC-5i and MDA-MB-231 cells was conducted after incubating the cells at 37°C for 1 h with BBB peptide shuttles and ACP at a final concentration of 10 μM, respectively. Blue is Hoechst 33342 (nucleus) and green is the derivatives. Scale bar = 20 μm. The fluorescence intensity of different images was determined to compare the fluorescence intensity of different fluorophores. This analysis was performed by calculating the corrected total cell fluorescence (CTCF).

The incubation of both BBB peptide shuttles with HBEC-5i shows that all derivatives are internalized (Figure 5). Thus, it indicates that there are no interference in cellular uptake. The fluorophore showing the highest background was Q570. Considering both AMPs incubated with MDA-MB-231 cells, the results are similar. All derivatives show high internalization capacity (Figure 5). Similarly to both BBB peptide shuttles, Q570 was the fluorophore showing the highest background.

The optimization of both the sensitivity of the detector and the laser intensity are also parameters to consider for each fluorophore. The derivatives that require the use of a more sensitive detector and a higher laser intensity were the CF-peptides. In addition, the exposition time was short, owing to the low photostability of the fluorophore. The use of RhB-peptides also requires some fine adjustments. Although it has a higher signal intensity, which allows the use of less sensitive detectors, the detection of RhB peptides requires the use of medium laser intensities. The use of CF and RhB is widely applied in research, mostly owing to their price. The use of highly advanced microscopes allows the detection of CF- or RhB-compounds. The use of either Q570-peptides or TF3-peptides overcome the previous limitations reported. Both fluorophores possess high signal intensities even at low laser intensities or using a less sensitive detector. In the absence of a high sensitive microspore, the use of these fluorophores might be highly advantageous. In addition, all the derivatives demonstrate stability at long exposition times.

## Conclusion

The study of peptides and the characterization of their potential biomedical application is limited to the capacity to visualize/quantify these peptides in cells. To do so, researchers rely on the use of fluorescent-labeled peptides to perform high sensitive techniques. However, until recently, very little was known about the influence of the fluorophore on the outcome of a given technique. The choice of the fluorescent probe was empirical and mainly based on spectral properties. In the present work, we selected four commercially available and highly used fluorophores with different physicochemical properties. Then, we conjugated them to four different peptides comprising two of the most important peptide applications, namely, BBB peptide shuttles and AMPs, which were tested as ACPs in this study owing to the high fraction of AMPs with anticancer activity.

Our results show that, indeed, fluorophores have an impact on peptide activity/toxicity depending on the peptide (Table 1). In general, the main characteristics of fluorophore groups are:

**TABLE 1:** Compilation of the results obtained with all peptides in different assay.

**Peptide**	**Fluorophore**	**Assay**													**Conclusion**
		**λ_exc_/λ_em_**	**Secondary Structure**	**Hemolytic activity (μM)**			**Translocation (%)**	**Cytotoxicity (μM)**						**Cellular imaging**	
								**MDA-MB-231 cells**			**HBEC-5i cells**				
				**HC_10_**	**HC_50_**	**HC_90_**		**IC_10_**	**IC_50_**	**IC_90_**	**IC_10_**	**IC_50_**	**IC_90_**		
PepH3	–	N.A.	Random coil	>200	>200	>200	55.15 ± 3.16	N.A.	N.A.	N.A.	>200	>200	>200	N.A.	The conjugation to RhB increases hemolysis and slightly decreases translocation
	5(6)-carboxy fluorescein	496/522		>200	>200	>200	57.09 ± 2.43	N.A.	N.A.	N.A.	>200	>200	>200	Low signal	
	Rhodamine B	568/598		9.208 ± 1.564	128.3 ± 6.60	>200	51.57 ± 4.81	N.A.	N.A.	N.A.	>200	>200	>200	Medium signal	
	Quasar 570	546/566		>200	>200	>200	59.60 ± 2.64	N.A.	N.A.	N.A.	>200	>200	>200	High signal Few background	
	Tide Fluor 3	558/588		>200	>200	>200	56.74 ± 3.89	N.A.	N.A.	N.A.	184.9 ± 6.57	>200	>200	High signal	
PepNeg	–	N.A.	Random coil	>200	>200	>200	50.16 ± 3.13	N.A.	N.A.	N.A.	>200	>200	>200	N.A.	The conjugation to Q570 slightly decreases translocation
	5(6)-carboxy fluorescein	496/522		>200	>200	>200	56.01 ± 3.49	N.A.	N.A.	N.A.	>200	>200	>200	Low signal	
	Rhodamine B	568/598		>200	>200	>200	51.57 ± 2.43	N.A.	N.A.	N.A.	>200	>200	>200	Medium signal	
	Quasar 570	546/558		>200	>200	>200	48.89 ± 3.05	N.A.	N.A.	N.A.	>200	>200	>200	High signal Few background	
	Tide Fluor 3	558/588		>200	>200	>200	53.69 ± 2.94	N.A.	N.A.	N.A.	>200	>200	>200	High signal	
vCPP2319	–	N.A.	Random coil	>200	>200	>200	N.A.	1.22 ± 0.52	5.02 ± 1.04	20.63 ± 2.103	N.A.	N.A.	N.A.	N.A.	The conjugation to RhB, Q570, and TF3 increases hemolysis and cytotoxicity toward MDA-MB-231 cells
	5(6)-carboxy fluorescein	498/530		>200	>200	>200	N.A.	1.26 ± 0.24	4.72 ± 0.94	18.76 ± 1.874	N.A.	N.A.	N.A.	Low signal	
	Rhodamine B	570/594		2.43 ± 0.32	23.88 ± 2.24	>200	N.A.	1.13 ± 0.64	4.11 ± 1.04	16.63 ± 1.312	N.A.	N.A.	N.A.	Medium signal	
	Quasar 570	546/568		7.79 ± 1.57	51.57 ± 4.61	>200	N.A.	1.14 ± 0.34	4.26 ± 1.03	16.00 ± 1.942	N.A.	N.A.	N.A.	High signal Few background	
	Tide Fluor 3	562/594		2.83 ± 0.97	>200	>200	N.A.	1.05 ± 0.21	4.30 ± 0.03	17.03 ± 1.582	N.A.	N.A.	N.A.	High signal	
Ctn[15-34]	–	N.A.	Random coil	>200	>200	>200	N.A.	4.76 ± 1.56	>200	>200	N.A.	N.A.	N.A.	N.A.	The conjugation to RhB and Q570 increases hemolysis; The conjugation to RhB, Q570, and TF3 increases cytotoxicity toward MDA-MB-231 cells
	5(6)-carboxy fluorescein	498/524		>200	>200	>200	N.A.	4.23 ± 1.02	>200	>200	N.A.	N.A.	N.A.	Low signal	
	Rhodamine B	564/598		3.29 ± 1.32	61.43 ± 4.33	>200	N.A.	2.97 ± 0.98	>200	>200	N.A.	N.A.	N.A.	Medium signal	
	Quasar 570	546/564		12.54 ± 3.26	193.8 ± 8.70	>200	N.A.	3.08 ± 1.22	144.0 ± 4.21	>200	N.A.	N.A.	N.A.	High signal Few background	
	Tide Fluor 3	558/588		102.0 ± 6.20	>200	>200	N.A.	2.57 ± 1.37	122.7 ± 3.84	>200	N.A.	N.A.	N.A.	High signal	
Conclusion		No differences	No differences	RhB, Q570, and TF3 decreases HC_10_	RhB and Q570 decreases HC_50_	No differences	No differences	RhB, Q570, and TF3 decreases IC_10_ of Ctn[15-34]	RhB, Q570, and TF3 decreases IC_50_ of vCPP2319 Q570 and TF3 decreases IC_50_ of Ctn[15-34]	RhB, Q570, and TF3 decreases IC_90_ of vCPP2319	No differences	No differences	RhB and Q570, decreases IC_90_ of Ctn[15-34]	Q570 and TF3 have the highest signal; Q570 shows some background	RhB seems to interference the most with the results from different assays

*CF, 5(6)-carboxyfluorescein; HC_10_, HC_50_, HC_90_, concentration that induces hemolysis in 10, 50, and 90% of red blood cells, respectively; IC_10_, IC_50_, and IC_90_, concentration that causes cell death in 10, 50, and 90% of human cells, respectively; N.A., Not applicable; RhB, rhodamine B; TF3, tide fluor 3; Q570, quasar 570.*

• CF has medium hydrophobicity, no toxicity, does not interfere with peptides’ activity, but has low fluorescence intensity signal;
• RhB has high hydrophobicity, high toxicity (conjugated to PepH3, vCPP2319, and Ctn[15-34]), decreases IC_10_, IC_50_, and IC_90_ in cancer cells, and medium fluorescence intensity signal;
• Q570 has high hydrophobicity, high toxicity (conjugated to vCPP2319, and Ctn[15-34]), decreases IC_10_, IC_50_, and IC_90_ in cancer cells, and high fluorescence intensity signal;
• TF3 has high hydrophobicity, and medium toxicity (conjugated to vCPP2319, and Ctn[15-34]), decreases IC_10_, IC_50_, and IC_90_ in cancer cells, and high fluorescence intensity signal.

Consequently, it is important to highlight that for low toxicity, the fluorescence intensity signal might be compromised. Overall, to select a fluorophore, it is necessary to consider the specific application/technique, since it can contribute to biased results.

## Data Availability Statement

All datasets presented in this study are included in the article/Supplementary Material.

## Author Contributions

VN, MAC, DA, and MC conceived and designed the experiments and wrote the manuscript with contribution from all other authors. MC, CP-P, JV, and RS performed the experiments. All authors contributed to the data analysis.

## Conflict of Interest

The authors declare that the research was conducted in the absence of any commercial or financial relationships that could be construed as a potential conflict of interest.
