# Erratum: To What Extent Do Fluorophores Bias the Biological Activity of Peptides? A Practical Approach Using Membrane-Active Peptides as Models

**DOI:** 10.3389/fbioe.2020.617198

**Published:** 2020-11-05

**Authors:** Frontiers Production Office

**Affiliations:** Frontiers Media SA, Lausanne, Switzerland

**Keywords:** anticancer peptides, BBB peptide shuttles, fluorescence, fluorophore, labeling

Due to a production error, the graphical abstract was removed from the final version of the article. The graphical abstract and its caption appear below.

**Graphical Abstract:**
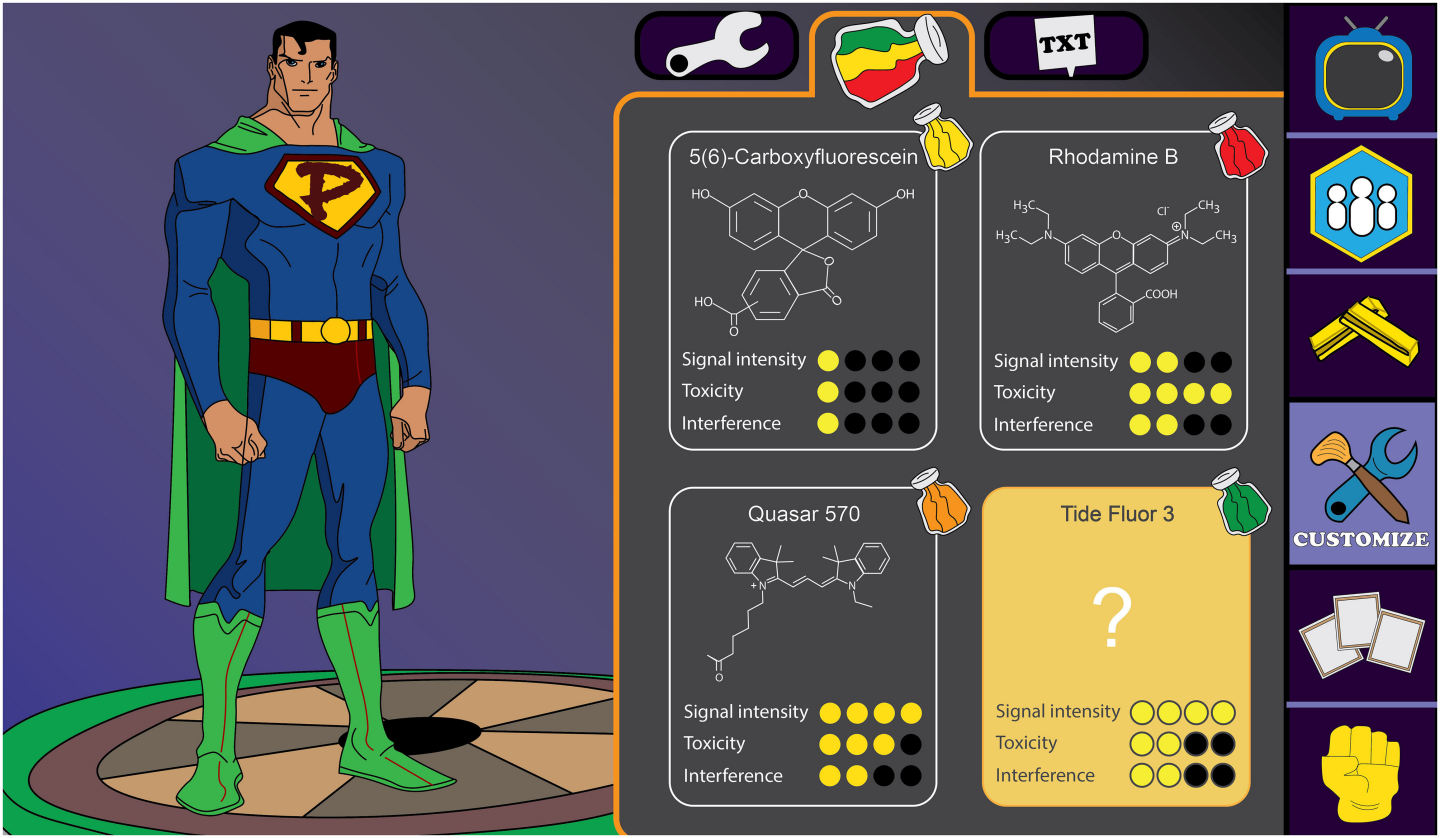
With this work we highlight the need for a careful experimental design when using fluorescently labeled molecules, since we show that some fluorophores significantly modulate peptide activity, which may contribute to biased results.

The publisher apologizes for this mistake. The original article has been updated.

