# Supplementary material for: To What Extent Do Fluorophores Bias the Biological Activity of Peptides? A Practical Approach Using Membrane-Active Peptides as Models
Source: Front Bioeng Biotechnol. 2020 Sep 9;8:552035. doi: 10.3389/fbioe.2020.552035 (PMC7509492; doi:10.3389/fbioe.2020.552035)
Supplement: Supplementary file 1 [file Data_Sheet_1.pdf]

Supplementary Material

Table S1. Properties of the selected fluorophores

**Fluorophore**	**Molecular weight (Da)**	**Charge**	**λ_{exc} (nm)**	**λ_{em} (nm)**	**Extinction coefficient ($M^{-1}.cm^{-1}$)**	**Quantum yield**	**Brightness[1]**	**logP[2]**	**pKa[2]**
5(6)-Carboxyfluorescein	376.32	-1	492	514	82,000	0.70	57,400	3.09	8.21
Rhodamine B	479.02	0	546	568	106,000	0.60	63,600	5.42	3.40
Quasar 570	471.65	+1	548	570	115,000	N.D.	N.D.	6.37	4.71
Tide Fluor 3	559.70	0	546	571	85,000	0.80	68,000	N.D.	N.D.

λ, wavelength; LogP, partition coefficient; N.D., not defined
[1]Calculated using the following formula: Extinction coefficient × Quantum yield
[2]Calculated using the Chem3D software (PekinElmer_Informatics)

PEKINELMER_INFORMATICS Chem3D. 19.1 ed.

Table S2. Properties of all selected peptides. The peptide sequence of each peptide was run in the Peptide Property Calculator (ver 3.1) (bioSYNTHESIS, 2013)

Application	Peptide	Physicochemical Properties				
		**Number of Residues**	**Net Charge**	**Extinction coefficient ($M^{-1}.cm^{-1}$)**	**Isoelectric point**	**Hydrophobicity (at pH 7.0)**
BBB peptide shuttle	PepH3	7	+2	5,690	14.00	42.86
	PepNeg	7	-2	1,280	4.15	-0.14
AMP	vCPP2319	20	+16	18,350	13.25	4.90
	Ctn[15-34]	20	+8	0	14.00	29.30

BIOSYNTHESIS 2013. Peptide Property Calculator.

Table S3. Peptides synthesized

Peptide	Amino acid sequence	Modification	Mass (Da), calculated (found)	HPLC t_R (min)[1]	Purity (%)
PepH3	AGILKRW-amide	none	842.8 (843.0)	5.5	99.5
	CF-AGILKRW-amide	Fluorophore	1,199.4 (1,201.4)	6.3	96.3
	RhB-AGILKRW-amide	Fluorophore	1,302.1 (1,303.7)	8.8	99.4
	Q570-AGILKRW-amide	Fluorophore	1,294.7 (1,295.6)	6.3	99.6
	TF3-AGILKRW-amide	Fluorophore	1,382.8 (1,382.6)	7.4	95.2
PepNeg	SGTQEEY-amide	none	811.8 (812.3)	4.5	99.5
	CF-SGTQEEY-amide	Fluorophore	1,169.1 (1,171.4)	3.8	99.1
	RhB-SGTQEEY-amide	Fluorophore	1,271.8 (1,272.0)	3.8	95.3
	Q570-SGTQEEY-amide	Fluorophore	1,264.4 (1,265.0)	5.6	99.6
	TF3-SGTQEEY-amide	Fluorophore	1,352.5 (1,353.1)	7.7	99.3
vCPP2319	WRRRYRRWRRRRRWRRRPRR-amide	none	3,177.8 (3,176.8)	6.9	99.1
	CF-WRRRYRRWRRRRRWRRRPRR-amide	Fluorophore	3,536.1 (3,537.8)	7.8	98.1
	RhB-WRRRYRRWRRRRRWRRRPRR-amide	Fluorophore	3,638.8 (3,639.4)	6.9	98.5
	Q570-WRRRYRRWRRRRRWRRRPRR-amide	Fluorophore	3,631.4 (3,631.8)	8.9	99.7
	TF3-WRRRYRRWRRRRRWRRRPRR-amide	Fluorophore	3,719.5 (3,719.6)	6.1	97.0
Ctn[15-34]	KKRLKKIFKKPMVIGVTIPF-amide	none	2,370.1 (2,369.5)	7.5	98.8
	CF-KKRLKKIFKKPMVIGVTIPF-amide	Fluorophore	2,728.4 (2,728.8)	7.2	96.6
	RhB-KKRLKKIFKKPMVIGVTIPF-amide	Fluorophore	2,831.1 (2,831.7)	7.8	99.1
	Q570-KKRLKKIFKKPMVIGVTIPF-amide	Fluorophore	2,823.8 (2,823.7)	11.9	96.4
	TF3-KKRLKKIFKKPMVIGVTIPF-amide	Fluorophore	2,911.8 (2,911.5)	6.2	95.2

[1]See experimental part for details

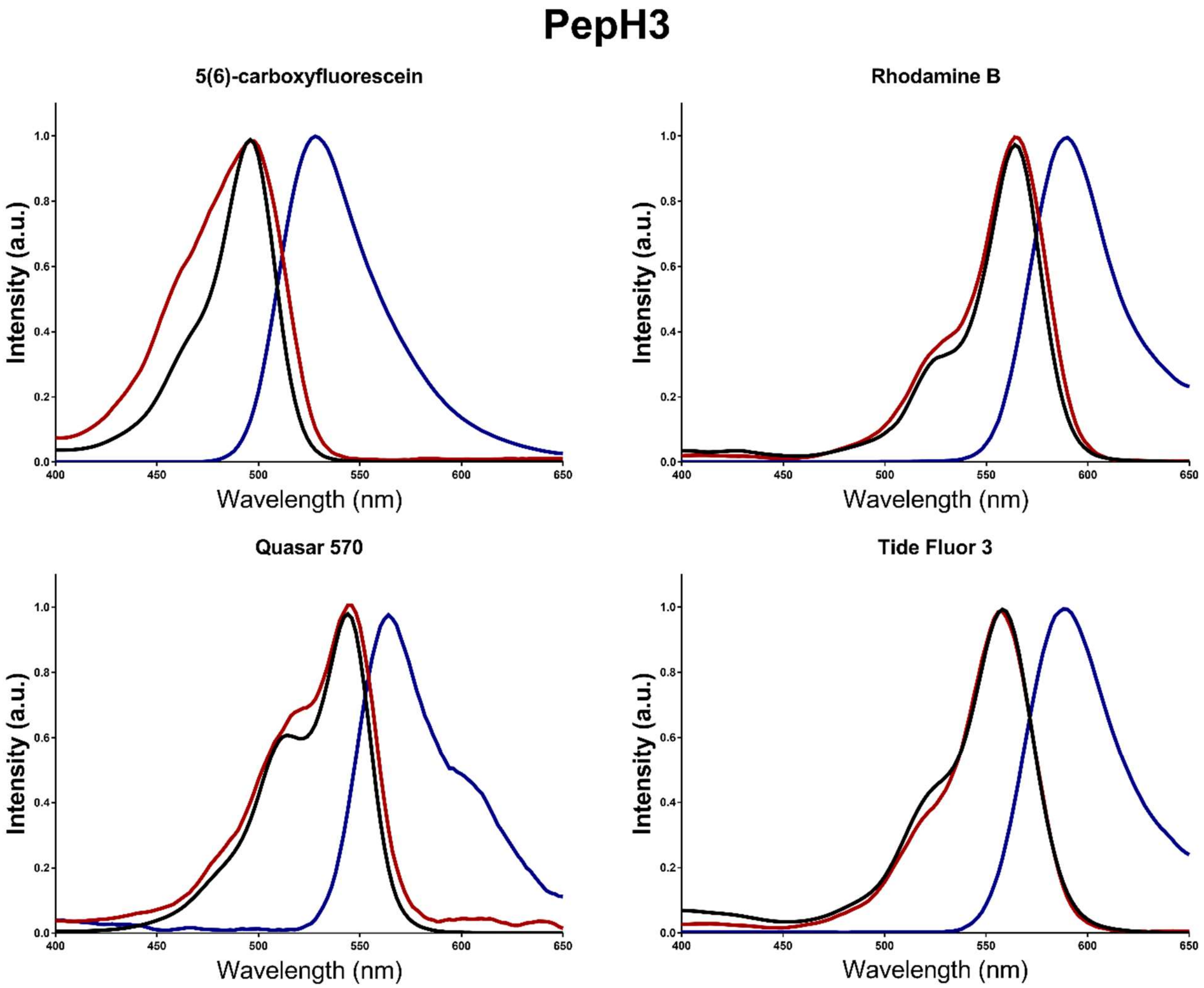

Figure S1 Spectrum of PepH3 derivatives. Black line – absorbance; red line – fluorescent excitation; and blue line – fluorescent emission.

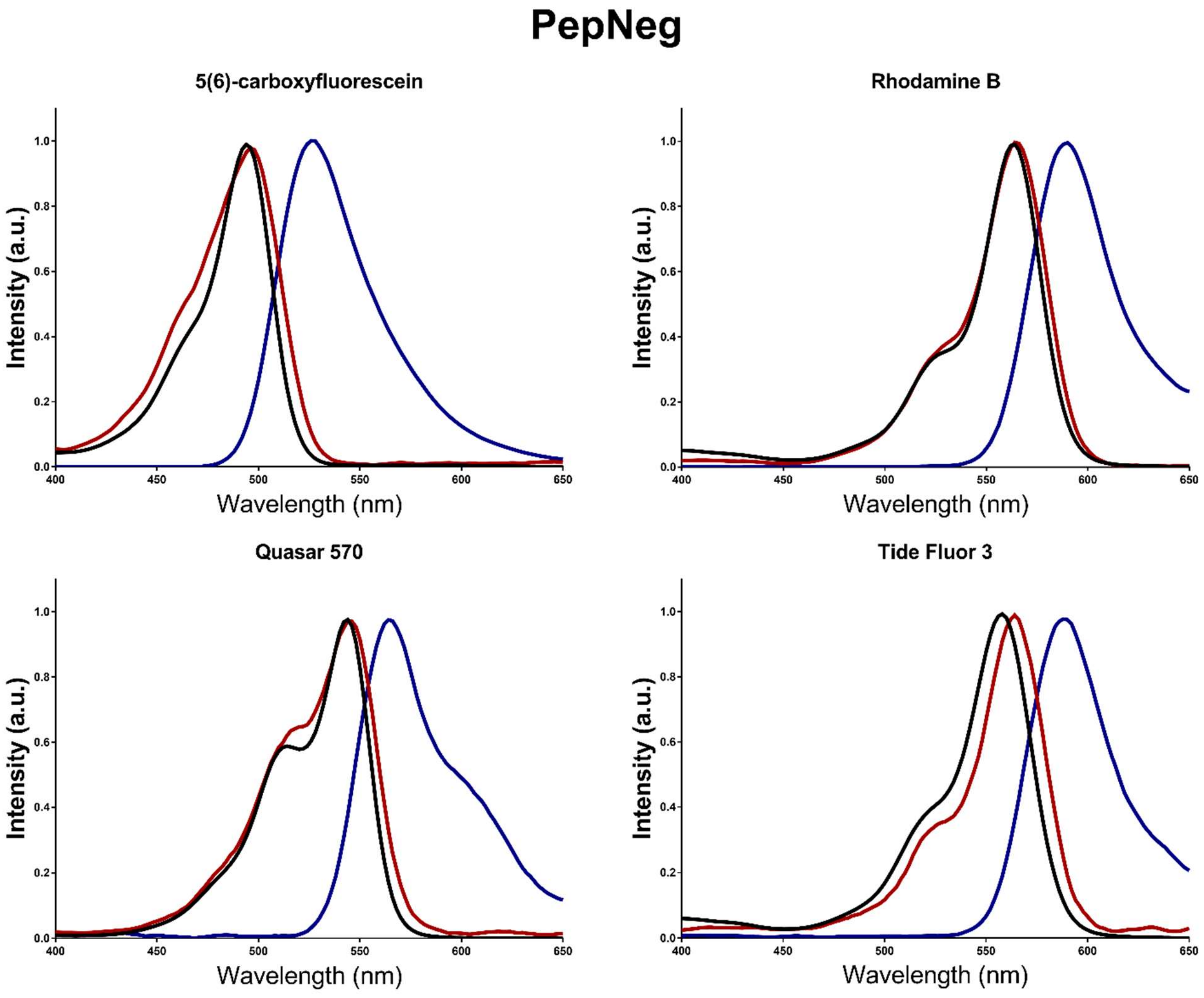

Figure S2 Spectrum of PepNeg derivatives. Black line – absorbance; red line – fluorescent excitation; and blue line – fluorescent emission.

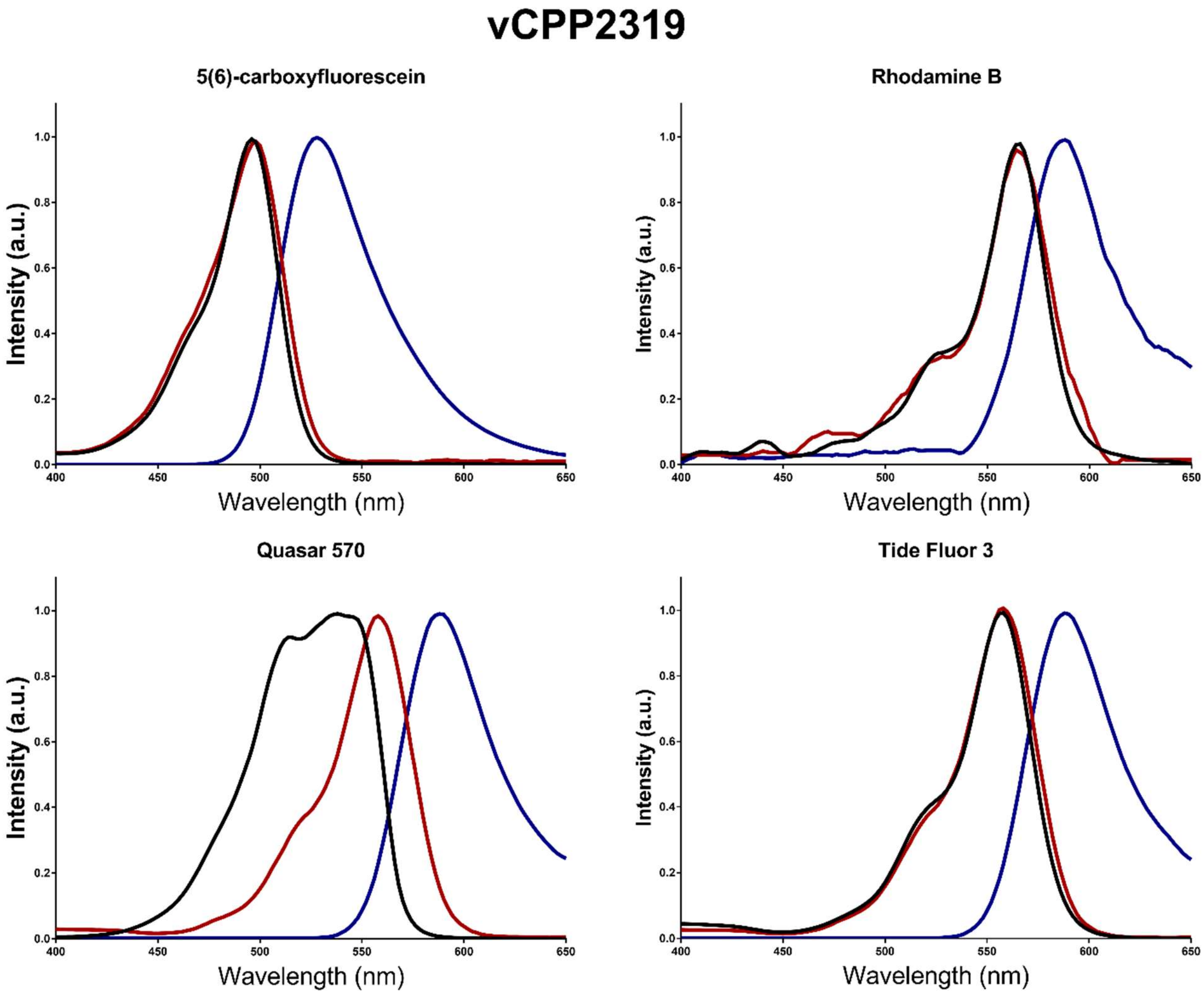

Figure S3 Spectrum of vCPP2319 derivatives. Black line – absorbance; red line – fluorescent excitation; and blue line – fluorescent emission.

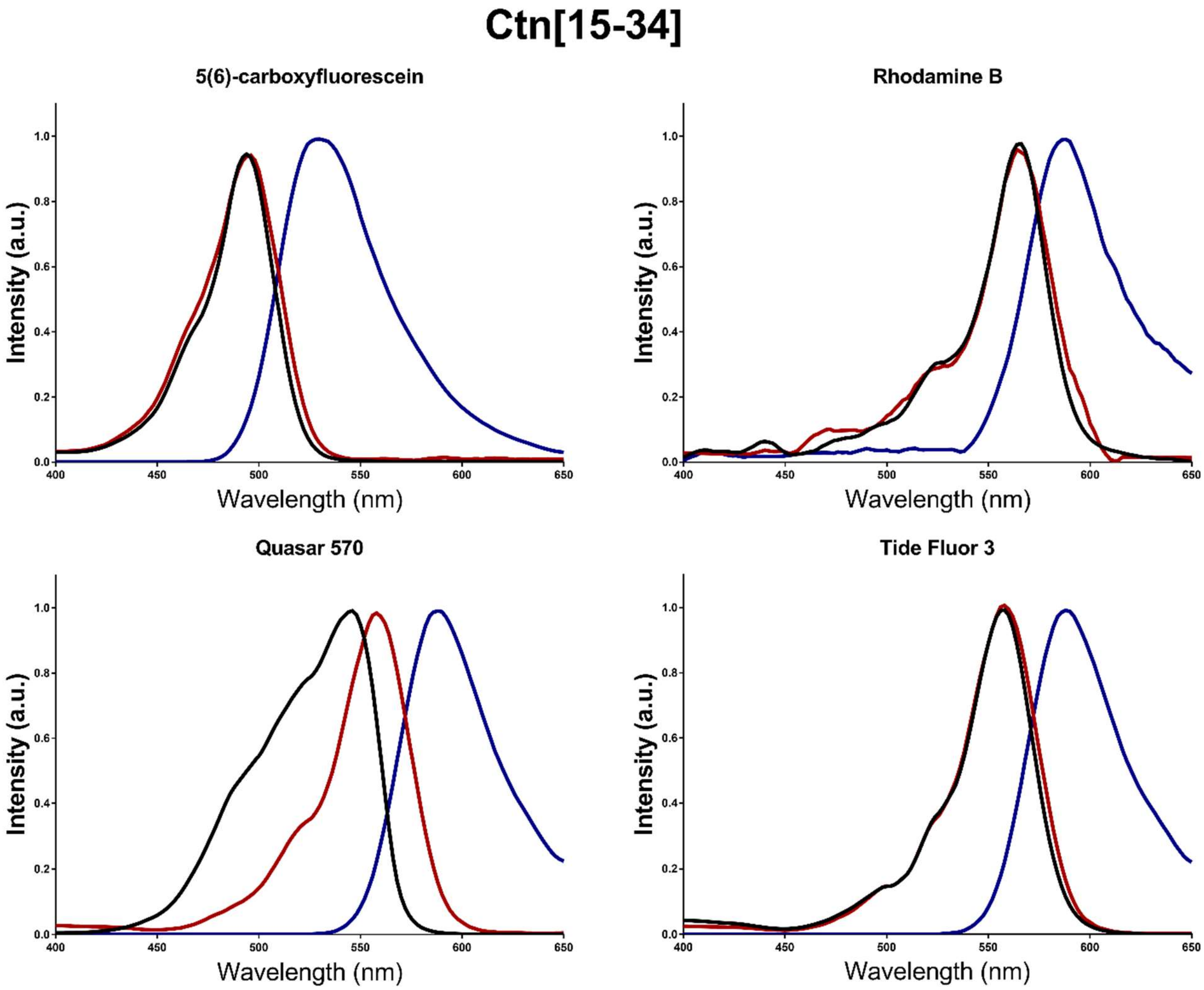

Figure S4 Spectrum of Ctn[15-34] derivatives. Black line – absorbance; red line – fluorescent excitation; and blue line – fluorescent emission.

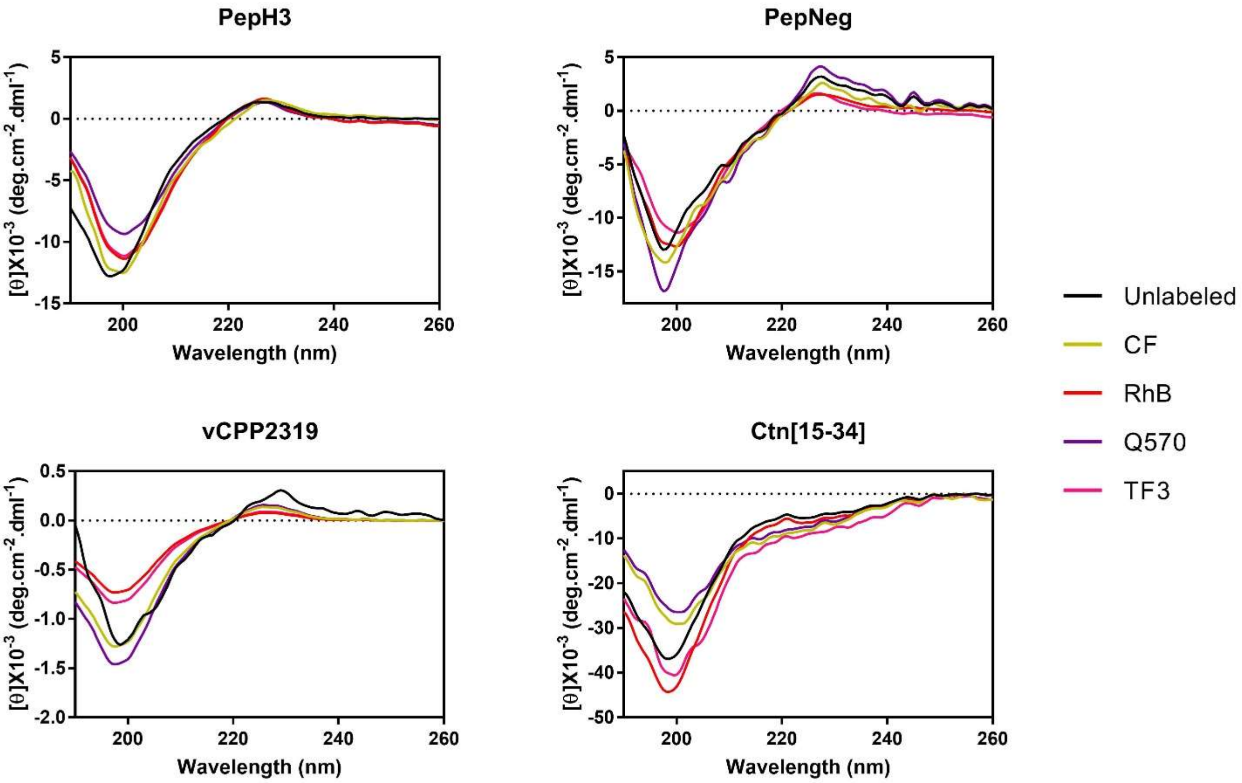

Figure S5 Secondary structure of unlabeled- and labeled-peptides. In phosphate buffer solution, the spectra of all peptides were characteristic of random-coil conformation. Upon derivation, no changes were observed.

Effect of fluorophores on the peptide structure

The secondary structure of peptides is important for their activity. (Sani and Separovic, 2018, Liu et al., 2018) Therefore, it is vital to understand the effect that a fluorophore might have on peptides secondary structure rearrangements. CD is a robust and reliable technique that allows this evaluation.(Bakshi et al., 2014, Greenfield, 2006) Thus, we performed CD to determine the influence of fluorophores on the secondary rearrangement of peptides. Firstly, we analyzed the conformation of unlabeled-peptides in phosphate buffer at 25°C. Then, we compared this conformation to the results obtained upon derivatization (Figure S5). Both unlabeled- and labeled-peptides show a random coil conformation. Thus, although some fluorophores are bulky and rigid, these results show that none of the fluorophores induces a conformational change in solution for peptides described as BBB peptide shuttles (PepH3 and PepNeg), or AMPs (vCPP2319 and Ctn[15-34]).

BAKSHI, K., LIYANAGE, M. R., VOLKIN, D. B. & MIDDAUGH, C. R. 2014. Circular Dichroism of Peptides. *In:* NIXON, A. E. (ed.) *Therapeutic Peptides: Methods and Protocols.* Totowa, NJ: Humana Press.

GREENFIELD, N. J. 2006. Using circular dichroism spectra to estimate protein secondary structure. *Nature protocols,* 1**,** 2876-2890.

LIU, S., BAO, J., LAO, X. & ZHENG, H. 2018. Novel 3D Structure Based Model for Activity Prediction and Design of Antimicrobial Peptides. *Scientific Reports,* 8**,** 11189.

SANI, M.-A. & SEPAROVIC, F. 2018. Antimicrobial Peptide Structures: From Model Membranes to Live Cells. *Chemistry – A European Journal,* 24**,** 286-291.

Table S4. Interaction of unlabeled- and labeled-peptides with RBCs. 1.0% (v/v) of RBCs were incubated with different concentrations of unlabeled- and labeled-peptides (range from 0.01 – 100 µM).

Peptide	Fluorophore	Hemolytic activity (HC50±SD µM)		
		HC_{10}	HC_{50}	HC_{90}
**PepH3**	-	>200	>200	>200
	5(6)-Carboxyfluorescein	>200	>200	>200
	Rhodamine B	9.208±1.564	128.3±6.596	>200
	Quasar 570	>200	>200	>200
	Tide Fluor 3	>200	>200	>200
**PepNeg**	-	>200	>200	>200
	5(6)-Carboxyfluorescein	>200	>200	>200
	Rhodamine B	>200	>200	>200
	Quasar 570	>200	>200	>200
	Tide Fluor 3	>200	>200	>200
**vCPP2319**	-	>200	>200	>200
	5(6)-Carboxyfluorescein	>200	>200	>200
	Rhodamine B	2.43±0.321	23.88±2.236	>200
	Quasar 570	7.79±1.569	51.57±4.612	>200
	Tide Fluor 3	2.83±0.969	>200	>200
**Ctn[15-34]**	-	>200	>200	>200
	5(6)-Carboxyfluorescein	>200	>200	>200
	Rhodamine B	3.29±1.321	61.43±4.326	>200
	Quasar 570	12.54±3.263	193.8±8.697	>200
	Tide Fluor 3	102.0±6.196	>200	>200
**Melittin**	-	0.16±0.025	0.78±0.354	3.84±0.961

HC_{10}, HC_{50}, and HC_{90} were defined as the concentration of peptide causing 10%, 50%, and 90% hemolysis on erythrocytes, respectively.

Table S5. Cytotoxicity of unlabeled- and labeled-peptides with human cells 1.5 x 10^4 and 1.0 x 10^4 cells of HBEC-5i and MDA-MB-231 cell lines, respectively, were incubated with different concentrations of unlabeled- and labeled-peptides (range from 0.01 – 100 μM).

Cell line	Peptide	Fluorophore	Cytotoxicity activity (IC50±SD μM)		
			IC_{10}	IC_{50}	IC_{90}
**HBEC-5i**	**PepH3**	-	>200	>200	>200
		5(6)-Carboxyfluorescein	>200	>200	>200
		Rhodamine B	>200	>200	>200
		Quasar 570	>200	>200	>200
		Tide Fluor 3	184.9±6.569	>200	>200
	**PepNeg**	-	>200	>200	>200
		5(6)-Carboxyfluorescein	>200	>200	>200
		Rhodamine B	>200	>200	>200
		Quasar 570	>200	>200	>200
		Tide Fluor 3	>200	>200	>200
**MDA-MB-231**	**vCPP2319**	-	1.22±0.521	5.02±1.041	20.63±2.103
		5(6)-Carboxyfluorescein	1.26±0.236	4.72±0.938	18.76±1.874
		Rhodamine B	1.13±0.641	4.11±1.045	16.63±1.312
		Quasar 570	1.14±0.345	4.26±1.027	16.00±1.942
		Tide Fluor 3	1.05±0.210	4.30±0.035	17.03±1.582
	**Ctn[15-34]**	-	4.76±1.564	>200	>200
		5(6)-Carboxyfluorescein	4.23±1.023	>200	>200
		Rhodamine B	2.97±0.984	>200	>200
		Quasar 570	3.08±1.221	144.0±4.213	>200
		Tide Fluor 3	2.57±1.369	122.7±3.845	>200

IC_{10}, IC_{50}, and IC_{90} were defined as the concentration of peptide causing 10%, 50%, and 90% death on human cells, respectively.
